# HIV infection and stroke in the Young in Abuja, Nigeria: a case series

**DOI:** 10.11604/pamj.2022.41.132.31270

**Published:** 2022-02-16

**Authors:** Henry Chijioke Onyegbutulem, Dilli Dogo, Aminu Mai, Theresa Chioma Obidike, Chinazo Sandra Ogbu-Eze, Amina Shehu, Ene Ebele Chidiebere, Charles Ezeude

**Affiliations:** 1 Department of Internal Medicine, Faculty of Clinical Sciences, College of Health Sciences, Nile University of Nigeria, Asokoro District Hospital, Abuja, Nigeria,; 2 Department of Surgery, Faculty of Clinical Sciences, College of Health Sciences, Nile University of Nigeria, Asokoro Hospital, Abuja, Nigeria,; 3 Department of Obstetrics and Gynecology, Faculty of Clinical Sciences, College of Health Sciences, Nile University of Nigeria, Asokoro District Hospital, Abuja, Nigeria,; 4 Department of Internal Medicine, Asokoro District Hospital, Abuja, Nigeria

**Keywords:** HIV-Positive, stroke in the young, Nigeria

## Abstract

Stroke is a major cause of disability and mortality among the Nigerian general population and thought to be commoner after the fifth decade of life and usually driven by conventional risk factors which are mainly cardio metabolic. However, with the youthful population in a city such as Abuja, stroke could be a mode of presentation of HIV in young people who are also more sexually active. Methods. This is a case series, reporting four cases of HIV positive young Nigerians with stroke. Patients´ data were retrieved from ward admissions records. The patients here had their socio-demographic data taken. They had presented with documented varied clinical features including those suggestive of stroke, after which they had HIV screening done which returned positive. One thousand four hundred and eighty-seven (1487) patients, were admitted in the medical ward, over a three-year period. Female to male ratio of 1:1 in the HIV-positive group, with an age range of 32 to 42 years and an average age of 37.5 years. Stroke constituted 5.7% of all admissions, with stroke in the young accounting for 1.2%. Of all stroke cases, stroke in the young constituted 21.43%, with those who were HIV positive accounting for 4.8%. Young people with stroke should be offered an HIV screening test.

## Introduction

Stroke is characterized as a neurologic deficit of vascular cause with an acute focal injury of the central nervous system (CNS) [1]. It is a major global cause of disability and death [1]. Generally, stroke affects both gender and while young people are not exempted, it is commoner after the fifth decade [2]. There are suggestions that HIV infection is associated with an increased risk of developing stroke in persons who are less than 45 years [3].

In sub-Saharan Africa (SSA), mostly in resource-poor regions, the extended family system prevails, thus stroke in the young is particularly tragic because most of the affected patients are in their most productive years of life with the likelihood of many responsibilities and dependents. This may create a long-term burden not only on the patients, but also on their immediate families and the wider community [4]. With reference to gender, there are suggestions that, HIV-positive women have an increased risk of cardiovascular disease (CVD), including ischaemic stroke, compared to HIV-negative women, although this diminishes with advancing age [5]. While the mechanisms behind this remain unclear, particularly in the young, HIV interacts with the host differently in women and in men. It is also possible that, endogenous sex hormones modify cardiovascular risk factors as well as pathways of immune activation and inflammation [5]. HIV is known to cause early menopause, which is associated with increased visceral fat, reduced muscle mass, and changes in bone density, which are all precursors to risk factors for cardiovascular diseases including stroke [5].

The Black race is more susceptible than Caucasians, partly because Blacks show more endothelial dysfunction when compared with other races, as well as differences in their genetic makeup [5]. Epidemiologically, the risk factors for stroke, particularly among the young in SSA, differ slightly from those in more advanced countries owing to differences in the environmental, behavioural and genetic factors between these populations [6]. While the young in the developed economy may have intravenous drug abuse as a risk factor, this is not exactly the same for most of Africa [6]. Stroke also affects a much younger patient population in low-income countries, than in high income countries [2].

Since over two-thirds of Africa´s HIV population live in SSA, it is expected that the burden of stroke among people living with HIV (PLWH), will be higher in this region [7]. A Malawian study found that the second most common cause of stroke was HIV [8]. A hospital-based study in Tanzania showed that, 20% of patients who presented with stroke were either diagnosed or already known to be living with HIV [9]. In regions with high HIV prevalence, studies have reported as high as 25% of its stroke cases as occurring in the young [9]. In these settings, most of the stroke risks in the young were attributable to HIV infection and its related factors [8, 9]. HIV infection significantly and independently increase stroke risk through some possible mechanisms´ viz; HIV-induced coagulopathy, vasculitis due to opportunistic infections, HIV-related intra- and extra-cranial vasculopathy and HIV induced cardiomyopathy [7-9]. Others are; the inflammation and immune activation associated with chronic HIV-infection and effects of antiretroviral therapy (ART) as well as decrease in proteins C and S levels [10].

The increase in antiretroviral therapy (ART) uptake, especially through the current test-and-treat approach as a national policy in Nigeria, and the decentralized models of care such as community antiretroviral group, CAG [11] should reduce morbidity and mortality from HIV Dolutegravir/AIDS and significantly increase the life expectancy. This consequent improvement in life expectancy has the potential of shifting the paradigm from just prevention/treatment/care to include focus on non-AIDS related emerging conditions, stroke inclusive. The Protease inhibitors, (PIs), have been implicated in CVD due to their association with dyslipidemia, lipodystrophy, and metabolic syndrome [12]. With the current common regime, Tenofovir, Lamivudine and Dolutegravir combo (TLD), there are emerging suggestions that, Dolutegravir may increase cardiovascular risk [12], and by extension stroke risk.

Clinically, PLWH with stroke present similar to HIV-negative persons, with sudden onset of focal neurological deficits being the most common presentation. However, PLWH with profound immunocompromised state may present atypically with symptoms of altered mental status, acute loss of consciousness, fever, or stepwise focal neurological deficits occurring over hours to days [8]. Strokes in PLWH can be subclinical, as demonstrated by autopsy studies compared with clinical studies [8]. Stroke could be the initial presentation of HIV infection, and HIV screening is an important tool for stroke evaluation among other investigations [9].

There are reports of stroke in the young from north-western [4], south-eastern [13], south-southern [14] and south-western [15] parts of Nigeria, whereas there are no similar report available from Abuja, the Capital city of Nigeria located in the north central part of the country, even with its high youthful population (94% are 49 years and below) [16], rising cardiovascular risk profile [17] and an HIV prevalence of 1.5% [18]. The objectives of this study therefore are; to describe the clinical profiles of cases of stroke in the young who were HIV -positive at the Asokoro Hospital in Abuja; provide sensitizing information to raise the index of suspicion of health workers on possible HIV-association in cases of stroke in the young.

## Methods

**Study design:** for this report, the age range of 18 to 44 years was used in selecting patients. The patients reported here were seen by our team over a three-year period between January 2016 and December 2018. The documentation was done by our team where patients were managed, particularly before the commencement of the test-and-treat policy. Relevant information was retrieved from the patients´ medical records, which were retraced using the ward admission register. The pages of the medical ward register from the 1^st^ of January 2016 to the 31^st^ of December 2018 were examined. All the ward admissions during that period were counted. All the stroke cases were sorted out, followed by selecting those between the ages of 18 and 44 years. Thereafter, those with documented HIV status were retrieved and only those who were HIV positive were reported. The medical case files of this small group were retrieved, and the needed data extracted accordingly. Because the number was small, the retrieved data were documented on Microsoft Word.

**Study setting:** this study took place at the Asokoro District Hospital, (ADH), a hundred and fifty-four bed capacity tertiary and teaching facility of the Nile University of Nigeria, in Abuja, the Capital city of Nigeria. Abuja is located in the centre of Nigeria, with a current population estimate of 3.5 million inhabitants. ADH provides services in all major medical specialties with a cumulative fourteen thousand (14,000), HIV-positive patients on records, with about five thousand, (5,600), being currently active.

### Participants

**Sample size:** the sample size was four HIV-positive patients with stroke who were between the ages of 18 and 44 years. Study participants were all the young patients who had stroke and were HIV positive. They were selected from all the stroke cases who were admitted and managed in the medical wards over the three-year review period.

**Variables and data source:** data retrieved from patients´ records include; demographics such as age, sex, marital status. Also, brief history, particularly the presenting complaints of unilateral paresis or paralysis. Past medical history to elicit the conventional risk factors. Social history included cigarette smoking, alcohol consumption and use of illicit drugs. History of multiple sexual partners as well. General and specific examination findings were retrieved, particularly, pallor, muscle power, blood pressure and presence of cardiac murmurs. Laboratory report of HIV status, chest X-ray, cranial CT scan, renal function test, full blood count, lipids and blood glucose were available. Treatment offered, include focused emergency care, ART and follow up at the out patients´ services

**Bias:** bias was eliminated by going through the ward admission register to check the diagnosis of all admissions in to the medical ward before sorting out those who had stroke. From all stroke patients admitted during the review period, those between age 18 and 44 years were set aside. From this group, those with documented HIV-positive status were selected as the cases for this report.

**Quantitative variables:** the quantitative variables include; total number of patients admitted in the medical ward during the review period. Number of all stroke cases, admitted in the respective years of 2016, 2017 and 2018. HIV status of all stroke cases between the ages of 18 and 44 years admitted in the review period. Number of HIV-positive stroke cases who were females and males between ages of 18 and 44 years. The number of deaths in this series were sorted; number of single patients and number with multiple sexual partners as well as the number who consumed illicit drugs, took alcohol and smoked cigarette. Durations before hospital presentation after hemiparesis, were all documented.

**Statistical methods employed:** direct counting was done, while ratios and percentages were calculated as applicable. The average age was obtained by dividing the total of the ages by four.

**Cases (participants)**

**Participant 1:** Mrs. O. F, a 36-year-old married female police officer, admitted on account of a two-month history of recurrent fever and progressive weight loss. She had a weeping left facial vesicular rash and a day´s history of right sided weakness. Not a known hypertensive or diabetic patient, not a known case of haemoglobinopathy. She neither consumed illicit drugs nor smoked cigarettes, but took two bottles of Guinness stout weekly. No seizure disorders. Examination revealed a young woman with a left sided herpetic rash pustular, distributed over the ophthalmic region of the trigeminal nerve. At entry, she was not febrile (36.5°C). Temperature during admission ranged between 36.8 - 37. 8°C. She was anicteric but pale (PCV 26%), conscious with right hemiparesis, with power of 3 in the upper and lower limbs. Her blood pressure was 120/80mm/Hg, No cardiac murmurs. Apart from hepatomegaly of 3cm on abdominal examination, other examinations were not remarkable. Serology was positive for HIV-1 with a CD4 count of 180 cells/cm^3^. Her renal function was normal. Entry random blood glucose was 128 mg/dl (7.1mmol/l), normal repeat and normal plasma lipids. Her cranial CT, was reported as normal. A routine chest X-ray was not remarkable. Herpes was suspected. She had Acyclovir, and antibiotics for the super-added bacterial infection on the facial rash.

**Outcome:** had sustained clinical improvement, was enrolled for HIV treatment/care and being followed up at the out patients´ services.

**Participant 2:** Miss O.P, 32-year-old single seamstress admitted with recurrent fever of three months, cough, weight loss and then right sided weakness of one day. She was not a known patient with haemoglobinopathy, hypertension, or diabetes mellitus. No history of significant alcohol consumption, cigarette smoking or use of illicit drugs. She had recurrent diarrhoea for about two months with a positive history of multiple sexual partners. On admission, she had hyperpigmented skin, dehydrated, febrile to touch (temperature- 38°C), anicteric and pale (PCV 22%). There was right hemiparesis, with power of 4 in the right upper limb and 2 in the right lower limb, with chest signs of consolidation on the right side, confirmed on chest radiograph. There was hepatomegaly of 2-4cm, with a tipped spleen. Her blood pressure was 90/60 mmHg. There were hemic murmurs with tachycardia of 102bpm. Blood was sterile on culture. The random blood glucose was 76mg/dl, with normal plasma lipids profile. Aside a hyponatraemia of 131mmol/l her renal assessment was normal. A computerized tomography (CT) scan was not done for financial reasons. She had stroke care, rehydration and systematic antibiotics. A follow-up chest X-ray after antibiotics reveal underlined features suggestive of tuberculosis. Even though she had a smear-negative sputum examination, her genexpert examination was positive.

**Outcome:** she improved remarkably, had anti tuberculosis treatment, and was enrolled in the adult HIV services and had ART.

**Participant 3:** A.A, a 42-year-old married commercial bus driver admitted with a two-day history of recurrent focal seizures involving the left lower limb. He was brought in with an altered level of consciousness on the second day. No history of long-standing fever or weight loss. He was not a previously known patient with seizure disorder, head injury, and was not a known hypertensive or diabetic patient. He smoked several sticks of cigarette and consumed large amounts of alcohol daily. At entry, he was restless, with a Glasgow Coma Scale (GCS) of 8/15, afebrile to touch, not pale (PCV 36%) and anicteric. There were bilateral planter blisters sustained from the use of hot plates to stimulate him after episodes of seizures at home. There was left hemiparesis, power was 2 in the right upper and lower limbs with facial nerve palsy. The chest was clear. The blood pressure was 130/70mm/Hg, no heart murmurs. Abdominal examination was unremarkable. He tested positive for HIV 1. The random blood glucose was 110mm/dl (6.1mmol/l). Cranial CT and other requested investigations were not done for financial reasons. He was commenced on parental anticonvulsant and cerebral decompression with minimal improvement.

**Outcome:** relatives requested for transfer to another facility for proximity and financial reasons.

**Participant 4:** A.O, a 40 -year old married businessman who was a known HIV-Positive patient already on ART (Truvada and Efavirenze) from the source of referral. He presented five hours after observing a progressive left sided weakness. He was not a known hypertensive patient, but a known diabetic patient who was on metformin for the previous two years. The patient did not smoke, but took alcohol occasionally. At entry, he was afebrile to touch, pale but anicteric. He was fully conscious and talking rationally. Power was 3 in the left upper limb and 4 in the left lower limb. Abdominal examination was unremarkable and safe for truncal obesity. Significant results of investigations done were: PCV of 28%, elevated total cholesterol, reduced high density lipoprotein, HDL and elevated low-density lipoprotein, LDL. The random blood glucose at entry was 142 mg/dl. Fasting and two hours post breakfast done the following day were; 5.3 mmol/l and 8.3 mmol/l respectively. Cranial CT confirmed ischaemic stroke. He had stroke care, vitamin E, C, as well as Aspirin. His diabetes control was ensured.

**Outcome:** he improved remarkably with physiotherapy, but was lost to follow up.

## Results

One thousand four hundred and eighty-seven (1487) patients, were admitted in the medical ward during the review period. Table 1 shows the total number of patients with corresponding number of stroke cases, admitted in the respective years of 2016, 2017 and 2018. There were two females and two males (1: 1 ratio) in the HIV-positive group, (in bracket), with an age range of 32 to 42 years and an average age of 37.5 years. Table 2 shows the socio-demographics of the HIV-positive cases reported here.

**Table 1:** number of admissions and cases of stroke in the Young HIV-Positive and HIV-Negative (in brackets)

	Total admissions	Total stroke cases			18-45yrs	>46yrs
Year	N (%)	N (%)	Male	Females		
2016	353 (23.74)	29 (34.5)	19	10	1 (5)	24
2017	350 (23.54)	19 (22.6)	7	12	1 (4)	15
2018	784 (52.72)	36 (42.9)	34	2	2 (9)	27
**Total**	**1487 (100.00)**	**8 4(100.0)**	**60**	**24**	**4 (18)**	**66**

**Table 2:** socio-demographics of the reported cases

	Case 1	Case 2	Case 3	Case 4
Age(years)	36	32	42	40
Sex	Female	Female	Male	Male
SEC	Lower	Lower	Lower	Middle
Drugs	-	-	-	-
Alcohol	Yes	-	Yes	Yes
Cigarette	-	-	Yes	-
Marital Status	Married	Single	Married	Married
MSP	-	Yes	-	-

SEC: Socioeconomic class. MSP: Multiple sexual partners. Drugs: meaning Illicit drugs

Table 3 displays some clinical and laboratory features of the reported cases. Stroke constituted 5.7% of all admissions, with stroke in the young accounting for 1.2%. Of all stroke cases, stroke in the young constituted 21.43%, with those who were HIV positive accounting for 4.8%. No deaths occurred in our series, even though we could not fully account for the outcome of case 3 who was referred on request.

**Table 3:** certain clinical and laboratory characteristics of the reported cases

	Case 1	Case 2	Case 3	Case 4
Hemiparesis	Yes	Yes	Yes	Yes
Fever	Yes	Yes	-	-
Weight loss	Yes	Yes	-	-
Diarrhoea	-	Yes	-	-
Duration before presentation	1 day	2 days	1 day	5 hrs
Seizures	-	Yes	Yes	-
Cough	-	Yes	-	-
Pallor	Yes	Yes	-	-
HTN	-	-	-	-
DM	-	-	-	Yes
Haemoglobinopathy	-	-	-	-
Opportunistic infection	Herpes	Tb	-	-
Skin	-	HPM	-	-
Sepsis	-	Yes	-	Yes
PCV (%)	26	22	36	28
CD4 (cells/mm3)	180	132	270	426
CT	Normal	Not done	Not done	Ischaemic

DM-Diabetes Mellitus; HTN-Hypertension; Tb-Tuberculosis; HPM-Hyperpigmentation; CT-Computerized Tomography; PCV-Packed cell volume.

One case who was single also had multiple sexual partners. Seventy-five percent of cases in this series were from the low socio-economic (SEC) background. Illicit drug use was not observed in any of the cases. Alcohol and cigarette consumption were found in 75% and 25% of the cases, respectively. Haemoglobinopathy, hypertension and heart diseases as risk factors were not found in this series. One patient who was previously known HIV positive also had diabetes mellitus and dyslipidaemia. One patient, case 2, (25%) had chronic cough which turned out to be tuberculosis and the same patient had chronic diarrhoea with risk of dehydration. Observed skin changes include herpetic rash in case 1, and hyperpigmentation in case 2. The case 2 also had hyponatremia. Altered level of consciousness (25%) and focal seizures (25%) were other forms of neurologic manifestations found aside hemiparesis. Two patients each had right and left hemiparesis respectively (1: 1 ratio). Durations before presentation after hemiparesis ranged from five hours to two days.

## Discussion

Globally, hypertension is a major risk factor for stroke [19], with HIV infection being an emerging unconventional risk factor from clinical and autopsy studies [8]. The average age of subjects in our study, was lower than the mean age for young people with stroke in the south-eastern parts of Nigeria [13], and higher than that reported in the northwestern parts of the country [4], as well as from an earlier study by Sarfo *et al*. among young West Africans with stroke [20]. This may be explained by the generally lower average age of the Abuja population [16]. Stroke in the young typically was shown to be commoner in the Abuja area of the country than in the southeast,15.5% and south-south, 8.8%, respectively [13, 14]. Even though the Abuja population is relatively younger, our small sample size could have placed a bias enough to explain this pattern. Interestingly, the HIV positivity and possibly HIV-related stroke in our series was lower than findings from earlier studies in the northwest and south-south parts of Nigeria [4, 14]. This is against the relatively higher HIV prevalence in the Abuja region. This may be partly explained by our small sample size. Furthermore, six young patients who had stroke were excluded from this report for lack of an HIV test result.

All the four cases had hemiparesis as a common feature, and this being a hallmark of stroke, is independent of HIV status. People living with HIV, PLWH, who are profoundly immunocompromised may present atypically with symptoms of altered mental status, acute loss of consciousness, fever, or stepwise focal neurological deficits occurring over hours to days [8]. One of our patients presented with progressive weakness, while another had altered level of consciousness and seizures.

Fever was a prominent feature among the cases in this series. Fever may be due to the HIV infection itself, bacterial infection as in case 2, opportunistic infection as in cases 1 and 2, and chronic inflammation [21]. Case 1 had herpes infection as an opportunistic infection. This manifested on the left side of the face, and the patient had right hemiparesis. This may be due to herpes-induced vasculitis extending to involve the intracranial vessels on the left side causing vasculitis which is a recognized mechanism for stroke in HIV-positive patients [21]. Also, some opportunistic infections such as tuberculosis are known to cause meningitis, which may be complicated by vasculitis and consequent constriction of the vessels running through exudes at the base of the brain. This may result in vasospasm and possible thrombosis [22]. Furthermore, chronic meningeal inflammatory exudates involving the adventitia may spread through the entire vessel wall, causing necrotizing panarteritis with secondary thrombosis and vessel occlusion [22].

A novel association with stroke risk among HIV patients is HIV-related dilated cardiomyopathy. None of our patients in this series had obvious cardiomegaly or heart disease. A previous Nigerian study reported existing cardiac diseases as attributable cause of stroke in 2.5% of stroke in the young, irrespective of HIV status [4]. HIV-related cardiomyopathy may predispose to cardiac thrombi, which may embolize. Also, endocarditis with vegetations may embolize to cause bleeding myotic aneurysms with haemorrhagic stroke. Left ventricular hypertrophy, LVH, is an independent stroke risk factor, although it does not appear to be a major factor for stroke in the young HIV positive patient.

Interestingly, advanced HIV infection may cause cachexia and with repeated fluid loss from diarrhoea, vomiting and increased insensible water loss (from fever), they may become dehydrated with relative hypotension resulting in “watershed” cerebral infarction. This may partly explain the mechanism of stroke in case 2 of our series. The patient improved remarkably after rehydration and correction of electrolytes. This same patient was observed to have hyperpigmented skin and hyponatremia alongside hypotension. These features suggest adrenal insufficiency, which is a recognized endocrine manifestation of HIV infection, especially in those with tuberculosis [23].

Anaemia was a common finding in our case series. It is a common haematological association of HIV, and most of the studies from Nigeria have shown prevalence of more than 50% [24]. Three out of four of our patients were anaemic. Even though the patients here were few, it is consistent with findings from a previous south-eastern Nigerian study [24], but higher than that in another study from Keffi, which is a city about 69.5 kilometres south-east of Abuja. This difference may be attributed to different socio-demographic and nutritional factors. Keffi is suburban compared to Abuja, and they cultivate and eat plenty of cheap iron and folic acid-rich vegetables. Anaemia affects outcomes in HIV patients, even among those on Anti-retroviral treatment (ARTs) [24]. Haemoglobinopathy, an unconventional risk factor for stroke in the young, was absent in our series. A previous study from north-western part of Nigeria, reported a haemoglobinopathy prevalence of 2.8% among cases of stroke in the young irrespective of HIV status [4].

HIV positive patients have an increased predisposition to hypercoagulable state, partly because of the elevated plasma levels of endothelial cell products, including von Willebrand factor (vWF) and soluble thrombomodulin (sTM), both of which have been shown to relate with viral load, suggesting that, high viremia may increase stroke risk [8]. Also, studies have shown a relationship between stroke risk and deficiencies in protein C and S in HIV positive patients [10]. This pattern increases coagulopathy risk. However, levels of these proteins were not assessed in this series.

Stroke risk in the HIV positive may be influenced by coexistence of conventional and other unconventional risk factors. Case 4 was a previously known HIV-positive patient with reduced high-density lipoprotein cholesterol (HDL-c) and elevated low-density lipoprotein cholesterol (LDL-c) levels, with an increase in plasma triglyceride levels. This dyslipidaemia pattern is common among HIV-positive patients, even prior to developing AIDS [12]. Furthermore, the degree of viremia may correlate with levels of triglycerides [12]. The case 4, in our series, who was also a diabetic patient, had dyslipidaemia and was ART-experienced. Certain ARTs are said to be associated with increased insulin resistance and dysglycaemia [12]. Interestingly, chronic inflammation and immune activation in HIV-infection and direct effects of antiretroviral therapy (ART) are significant in cardiovascular risk profile partly through their damaging effect on the endothelium resulting in endothelial dysfunction [12]. Endothelial dysfunction, has been shown to play a role in HIV associated stroke risk, as demonstrated by an increased pulse wave velocity (which may be an early marker for atherosclerosis) in HIV positive patients [12]. The case 4 was on ARTs.

Available literature indicates that, most HIV-related strokes are ischaemic, and this is due to the nature of the predominant risk factors. The aetiology of ischaemic stroke in PLWH is multifaceted and can be grouped into several categories, including: large-artery atherosclerosis (LAA), small-vessel disease (SVD), cardio-embolism, and stroke due to other aetiologies, including infection-related strokes, coagulopathy and non-atherosclerotic HIV-associated vasculopathy, with no clear aetiology in some cases [10, 21]. Certain neoplastic conditions such as Kaposi sarcoma and primary CNS lymphoma are commoner in HIV-infected patients, and as intracranial space occupying lesion, SOL, they could mimic stroke in this group of patients [25] with stroke-like syndrome. Kaposi sarcoma tends to bleed and may mimic hemorrhagic stroke.

There was no mortality in our series, even though the outcome of case 3 could not be concluded in this write-up, since the patient who was very sick was referred out on request. Stroke in the young generally carries significant case fatality, as high as 10.5% through 16.7% to 23.9% in reports on previous Nigerian studies from the south-east, south-south and northwest respectively [4, 13, 14]. This is quite significant as it concerns young people whose viable prospects are threatened.

**Limitations:** this report was limited by our inability to carry out neuroimaging for some of our patients. This was primarily for financial reasons, which is prevalent in resource-poor settings. However, a good history and thorough clinical examination were helpful in assessment. Also, screening for some opportunistic infections and certain unconventional risk factors were not done. A large study, preferably multicentre, is recommended as this may relate markers of immunodeficiency such as CD4 count and viral load to stroke risk among young Nigerian HIV positive patients.

## Conclusion

Stroke in the young may be a mirror of a background HIV infection. It is therefore necessary to counsel young persons with stroke for HIV screening. This may offer an opportunity for better care that will also include early targeted HIV treatment and care, with a likely better outcome.

### What is known about this topic

- HIV infection is an unconventional risk factor for stroke majorly ischaemic;
- Young persons who are HIV positive are high risk for stroke;
- Certain anti-retroviral drugs predispose to stroke events in HIV positive who are treatment-experienced.

### What this study adds

- Anaemia among HIV positive patients is less common in a suburban part of Nigeria than in Abuja due to possible more consumption of iron-rich green leafy vegetables in this agrarian area;
- Adding the Abuja experience to available literature on stroke among young persons as related to HIV infection.
